# Optimization of Three Extraction Methods and Their Effect on the Structure and Antioxidant Activity of Polysaccharides in *Dendrobium huoshanense*

**DOI:** 10.3390/molecules28248019

**Published:** 2023-12-08

**Authors:** Hua Zhu, Xin Yi, Si-Si Jia, Chun-Yao Liu, Zi-Wei Han, Bang-Xing Han, Gong-Cheng Jiang, Zheng-Feng Ding, Ren-Lei Wang, Guang-Ping Lv

**Affiliations:** 1 School of Food and Pharmaceutical Engineering, Nanjing Normal University, Nanjing 210046, China; wfzh8233@163.com (H.Z.); 212702035@njnu.edu.cn (X.Y.); 18835540923@163.com (S.-S.J.); wissingni@163.com (C.-Y.L.); hinziwei@163.com (Z.-W.H.); 2 College of Biological and Pharmaceutical Engineering, West Anhui University, Lu’an 237012, China; 3 Key Laboratory of Biological Functional Molecules of Jiangsu Province, College of Life Science and Chemistry, Jiangsu Second Normal University, Nanjing 211200, China; ji680103@163.com (G.-C.J.); ding@jssnu.edu.cn (Z.-F.D.)

**Keywords:** *Dendrobium huoshanense*, polysaccharide, response surface methodology, structure characterization, antioxidant activity

## Abstract

*Dendrobium huoshanense* is a famous edible and medicinal herb, and polysaccharides are the main bioactive component in it. In this study, response surface methodology (RSM) combined with a Box–Behnken design (BBD) was used to optimize the enzyme-assisted extraction (EAE), ultrasound–microwave–assisted extraction (UMAE), and hot water extraction (HWE) conditions and obtain the polysaccharides named DHP-E, DHP-UM, and DHP-H. The effects of different extraction methods on the physicochemical properties, structure characteristics, and bioactivity of polysaccharides were compared. The differential thermogravimetric curves indicated that DHP-E showed a broader temperature range during thermal degradation compared with DHP-UM and DHP-H. The SEM results showed that DHP-E displayed an irregular granular structure, but DHP-UM and DHP-H were sponge-like. The results of absolute molecular weight indicated that polysaccharides with higher molecular weight detected in DHP-H and DHP-UM did not appear in DHP-E due to enzymatic degradation. The monosaccharide composition showed that DHPs were all composed of Man, Glc, and Gal but with different proportions. Finally, the glycosidic bond types, which have a significant effect on bioactivity, were decoded with methylation analysis. The results showed that DHPs contained four glycosidic bond types, including Glc*p*-(1→, →4)-Man*p*-(1→, →4)-Glc*p*-(1→, and →4,6)-Man*p*-(1→ with different ratios. Furthermore, DHP-E exhibited better DPPH and ABTS radical scavenging activities. These findings could provide scientific foundations for selecting appropriate extraction methods to obtain desired bioactivities for applications in the pharmaceutical and functional food industries.

## 1. Introduction

*Dendrobium* is a well-known edible and medicinal herb worldwide. However, there are many different species in the genus and their quality varies greatly [1]. In Pharmacopoeia of the People’s Republic of China, *Dendrobii Caulis* is officially documented as the fresh or dry stems of *D. huoshanense* C. Z. Tang et S. J. Cheng, *D. fimbriatum* Hook., *D. chrysotoxum* Lindl., and *D. nobile* Lindl. Among them, the resources of *D. huoshanense*, which is considered to be the most effective species [2], are the scarcest. Polysaccharides are the main bioactive component in *D. huoshanense*, and the content of water-soluble polysaccharides in *D. huoshanense* is mainly between 12% and 25%. Previous findings have proven that polysaccharides in *D. huoshanense* possess beneficial effects in alleviating gastric ulcers [3], osteogenesis promotion [4], hypoglycemic activities, etc. [5]. The polysaccharides with a backbone composed of →1)-Glc*p*-(4→ and →1)-Man*p*-(4→ and a backbone composed of →1)-Gal*p*-(4→, →1)-Glc*p*-(4→, →1)-Xyl*p*-(4→, and →1)-Glc*p*-(6→, etc., have been previously separated from *D. huoshanense* [2]. However, the overall view of the chemical composition of the polysaccharides in the water extract of *D. huoshanense* has not been decoded.

Furthermore, different extraction methods have a remarkable influence on the extraction efficiency, structure characteristics, and bioactivity of polysaccharides [6]. There are many methods that have been applied for the extraction of polysaccharides from natural sources, including enzyme-assisted extraction (EAE), microwave-assisted extraction (MAE), ultrasonic-assisted extraction (UAE), hot water extraction (HWE), etc. [7]. Among them, the HWE method is the most commonly used extraction method; UAE, MAE, and EAE are used to promote polysaccharide solubilization through the mechanical disruption or biodegradation of plant cell walls [8]. Research has shown that the MAE of *Potentilla anserina* polysaccharides reduces the molecular weight of polysaccharides compared with HWE, resulting in a hydrolytic cleavage of intermolecular hydrogen bonds and further enhancing antioxidant activity [9]. UAE-extracted polysaccharides contain higher levels of uronic acid, and their antioxidant activity is superior to that of other polysaccharides extracted with cold extraction (CE), HWE, and MAE [10]. In addition, there are complex interactions between different assisted extraction parameters, which can definitely affect the extraction efficacy. It was reported that a significant interaction was observed in the enzymatic ultrasound-assisted extraction (EUAE) of polysaccharides from *Momordica charantia*, and the interaction was evaluated with response surface methodology (RSM) combined with a Box-Behnken design (BBD) on extraction pH, temperature, and ultrasonic time [11]. However, after investigation, the effect of different extractions on the structural properties and bioactivities of *D. huoshanense* polysaccharides has never been studied, which is conducive to the industrial development and clinical application of *D. huoshanense*.

Therefore, commonly used assisted extraction methods including EAE and ultrasound-microwave-assisted extraction (UMAE) were compared with traditional HWE for polysaccharides in *D. huoshanense* in this study. Single factor test and BBD were applied for the optimization of the extraction condition. The polysaccharides extracted with three methods were systematically characterized and compared for their physicochemical properties, morphology, weight-average molecular weight (Mw), monosaccharide compositions, and glycosidic bond types with chemical analysis, X-ray diffraction analysis, thermal analysis, scanning electron microscopy (SEM), high-performance size-exclusion chromatography coupled with multi-angle laser light scattering and refractive index detector (HPSEC-MALLS-RID), gas chromatography-mass spectrometry (GC-MS), and methylation analysis. Furthermore, the antioxidant effects of the obtained polysaccharides were investigated by using the DPPH and ABTS radical scavenging models. The impact of the extraction method on the specific morphology, properties, structure characteristics, and bioactivity of polysaccharides elucidated in this study is beneficial to the utilization of natural polysaccharides.

## 2. Results and Discussion

### 2.1. Optimization of Enzyme-Assisted Extraction (EAE) of Polysaccharides from D. huoshanense

Enzyme-assisted extraction can promote the biodegradation of plant cell walls, thereby increasing the extraction efficiency of polysaccharides. The parameters which affect EAE efficiency, including the liquid-solid ratio, extraction pH, extraction temperature, and extraction time, were optimized with a single-factor test and response surface methodology combined with a Box-Behnken design. The optimization results are as follows:

#### 2.1.1. Optimization Results of EAE with Single-Factor Test

**Figure 1:**
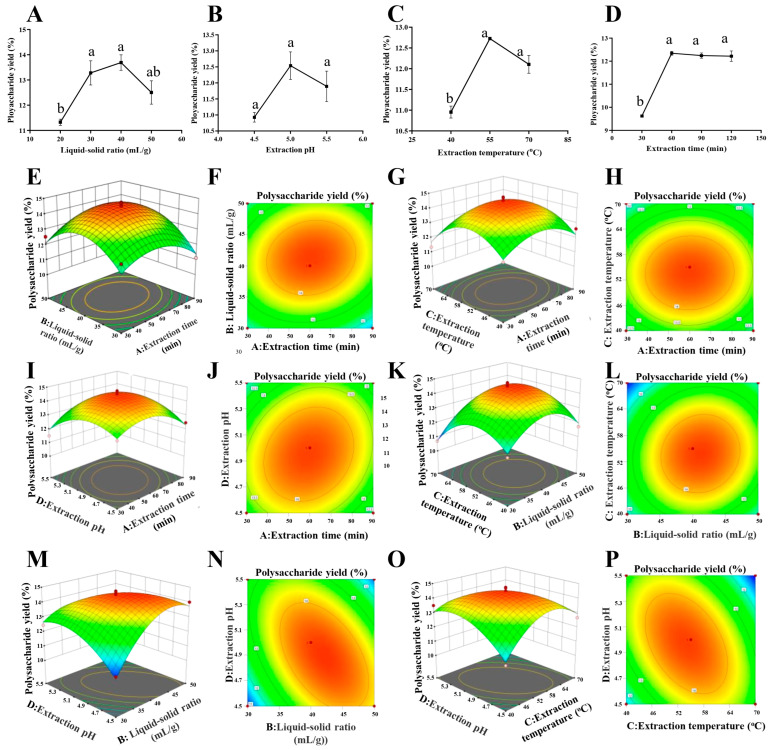
Effect of different (**A**) liquid–solid ratio, (**B**) extraction pH, (**C**) extraction temperature, and (**D**) extraction time on the yield of polysaccharides with EAE. Response surface plots (**E**,**G**,**I**,**K**,**M**,**O**) and contour plots (**F**,**H**,**J**,**L**,**N**,**P**) show the effects of liquid–solid ratio, extraction pH, extraction temperature, and extraction time on the yield of polysaccharides with EAE. Significant (*p* < 0.05) differences are shown by data bearing different letters (a,b).

The optimization results of EAE with the single-factor test are shown in Figure 1. Figure 1A shows that the liquid–solid ratio significantly affected the extraction efficacy. When the liquid–solid ratio was increased to 40:1 (mL/g), the yield of polysaccharides was highest, which was 13.69%. The yield of polysaccharides showed a decreased trend when the liquid–solid ratio was larger than 40:1 (mL/g). This is probably because a larger extraction volume undoubtedly increases the concentration time and leads to the degradation of polysaccharides. Finally, it results in a decreased trend in this study [12]. From Figure 1B, it can be seen that the extraction rate showed a significant increase with the increase in pH value, and at pH 5, the extraction rate was the highest, reaching 12.54%. A suitable pH value is beneficial for enzymes to maintain their spatial structure [13]. It is evident from Figure 1C that the yield of polysaccharides increased with the increasing extraction temperature until the yield reached a maximum value of 12.73% at 55 °C. A temperature higher than 55 °C probably reduces the activity of cellulase, so the polysaccharide extraction rate decreased. A previous study, which focused on cellulase-assisted polysaccharide extraction, investigated 40, 45, 50, 55, 60, and 65 °C for the extraction and found that the extract efficacy was significantly influenced by the temperature. The result confirmed that 55 °C is the best extraction temperature for cellulase-assisted polysaccharide extraction in *pumpkin (Cucurbita moschata)*, which supported the results of temperature optimized in this study [14]. From Figure 1D, it is evident that the yield of polysaccharides increases with the increase in extraction time and reaches a maximum value of 12.35% when the extraction time reaches 60 min. However, when the time of extraction was greater than 60 min, the yield of DHP was not significantly increased. Based on the observed maximum values of these factors, factors including the liquid–solid ratio (30:1, 40:1, 50:1 (mL/g)), extraction pH (4.5, 5.0, 5.5), extraction temperature (40, 55, 70 °C), and extraction time (30, 60, 90 min) were selected for BBD optimization to examine the interaction between factors (Table 1).

#### 2.1.2. Optimization Results of EAE with RSM with BBD

The results of the single-factor experiment indicate that the extraction of DHP-E is closely related to the extraction conditions. The parameters related to the extraction conditions are, to some extent, interrelated. The extraction process for polysaccharides exhibited a complex nature. Therefore, the RSM experimental design was combined with BBD to further explore the correlations between the factors in order to determine the optimal extraction conditions.

##### Model Fitting and Statistical Analysis of EAE

The experimental data of the BBD study were derived using regression analysis, employing a second-order polynomial equation:

Y = 14.48 − 0.0033 × A + 0.3608 × B − 0.2592 × C − 0.1833 × D + 0.295 × AB +  0.0625 × AC + 0.3775 × AD + 0.2625 × BC − 1.12 × BD − 0.9675 × CD − 1.01 ×  A^2^ − 1.43 × B^2^ − 1.46 × C^2^ − 0.9743 × D^2^ (1)

where Y is the yield of polysaccharides and A, B, C, and D are the coded values for extraction time, liquid–solid ratio, extraction temperature, and pH, respectively.

The detailed investigation of the variance and the accuracy of the models is summarized and presented in Table 2, from which it can be seen that the interaction terms BD and CD and the quadratic terms A^2^, B^2^, C^2^, and D^2^ had a highly significant effect on the extracted polysaccharides, while the effects of the other factors were not significant. The order of the factors affecting the yield of polysaccharides was obtained as follows: liquid–solid ratio > temperature > pH > time. This model has a *p* < 0.0001, and the response surface regression model achieved a highly significant level of statistical significance (*p* < 0.01). Additionally, the lack-of-fit test yielded a non-significant result (*p* = 0.2324 > 0.05), indicating that the model adequately captured the data. The coefficient of variation (C.V.) was found to be 3.34%, which falls below the threshold of 10%. This signifies that any untested factors had minimal influence on the outcomes, affirming the model’s strong experimental stability. The model correlation coefficient R^2^ was 0.9414, which indicates that the experimental model fits well with the actual test [15]. The calibrated coefficient of determination R^2^_Adj_ was 0.8829, which was close to R^2^, demonstrating that the model exhibits substantial accuracy and versatility [15]. Consequently, it can effectively serve as a tool for analyzing and predicting the impacts of various factors on the extraction rates in the EAE process.

##### Response Surface Analysis of EAE

The two-dimensional (2D) contour plots and three-dimensional (3D) response surfaces are shown in Figure 1E–P. The three-dimensional response surface visualizes the regression equation, and thus, the interaction between any two variables and the relationship between the test variable and the response could be evaluated.

In the 2D contour plots, elliptical contour plots indicate significant interactions between variables. In contrast, circular contour plots indicate negligible interactions [15].

Figure 1M,N indicate that the yield of polysaccharides obtained first increases and then decreases with the increase in pH in the case of a low liquid–solid ratio. Moreover, the high inclination and steep slope of the response surface indicate the highly significant interaction of the liquid–solid ratio and pH. Similarly, Figure 1O,P show that the interaction of the extraction temperature and pH has a significant effect on the extracted DHP. On the contrary, Figure 1E–L indicate that they did not interact significantly.

##### Verification of Predictive Model of EAE

The optimum DHP-E parameters are an extraction time of 33.9223 min, a liquid–feed ratio of 41.9729:1 (mL/g), an extraction temperature of 55.6157 °C, and a pH of 4.80166. The maximum yield was predicted to be 13.8217%. Considering the ease of operation during the extraction process, the extraction parameters were slightly adjusted: an extraction time of 34 min, a liquid–solid ratio of 42:1 (mL/g), an extraction temperature of 56 °C, and a pH of 4.8. In order to verify its feasibility, two groups of validation tests were conducted under this condition, and the extraction rate of DHP-E was 14.36 ± 0.20%. The relative error between the predicted values and actual measured values is 3.75%, which is mainly caused by the experimental errors and model errors, and it is acceptable according to previous studies [16,17,18]. The RSM model has been validated with the experimental results, suggesting that the model is valid for the DHP-E process.

### 2.2. Optimization of Ultrasound–Microwave-Assisted Extraction (UMAE) of Polysaccharides from D. huoshanense

Ultrasonic-assisted extraction (UAE) and microwave-assisted extraction (MAE) are commonly used to promote the dissolution of polysaccharides through the mechanical destruction of plant cell walls, especially microwaves, which are responsible for heating the moisture inside the cell, causing cell fragmentation, and increasing the dissolution of intracellular solutes. The parameters, including the liquid–solid ratio, ultrasonic time, ultrasonic temperature, microwave power, and microwave time, were optimized, and the results are as follows:

#### 2.2.1. Optimization Results of UMAE with Single-Factor Test

Figure S1 summarizes the optimization results of UMAE with a single-factor test. The yield of polysaccharides was optimized in UMAE with different liquid–solid ratios ranging from 20:1 to 40:1 (mL/g), as displayed in Figure S1A, and reached its maximum point at a liquid–solid ratio of 30:1 (mL/g). As shown in Figure S1B, the yield of DHP was significantly increased at the first stage of the ultrasound process (30–90 min), which may be attributed to the fact that the ultrasonic effect in the pre-extraction stage causes the release of cellular components by disrupting the plant cell wall [19]. As shown in Figure S1C, the yield of DHP exhibited a noteworthy enhancement as the ultrasonic temperature was elevated from 20 to 40 °C. Nevertheless, the yield of DHP did not significantly increase at 60 °C. As shown in Figure S1D, the yield increased when the microwave power was increased from 200 to 400 W. However, the yield of polysaccharides decreased when the microwave power was further increased. This is probably because excessive microwave intensity would result in the carbonization of polysaccharides, and we have also observed its related phenomena during the experimental process. As shown in Figure S1E, with an increase in microwave time longer than 4 min, the yield of DHP decreased significantly, indicating that a too long extraction time leads to the degradation of polysaccharides [20]. Therefore, based on the result of the single-factor test, the liquid–solid ratio (20:1, 30:1, 40:1 (mL/g)), ultrasonic time (60, 90, 120 min), ultrasonic temperature (20, 40, 60 °C), microwave power (200, 400, 600 W), and microwave extraction time (3, 4, 5 min) were selected to carry out BBD optimization, and the experimental design for the independent variables is shown in Table S1.

#### 2.2.2. Optimization Results of UMAE with RSM with Box–Behnken Design

##### Model Fitting and Statistical Analysis of UMAE

The second-order polynomial equation of UMAE is shown below:

Y = 11.75 + 0.8153 × A − 0.3256 × B + 0.1609 × C − 0.5288 × D − 0.4301 × E + 0.5175 × AB + 0.1875 × AC +  0.361 × AD − 0.0163 × AE + 0.9265 × BC + 0.7811 × BD − 0.095 × BE − 0.475 × CD − 0.3822 × CE + 0.795 × DE  − 0.3267 × A^2^ − 1.09 × B^2^ − 0.8819 × C^2^ − 1.17 × D^2^ − 0.7566 × E^2^ (2)

where Y is the yield of polysaccharides and A, B, C, D, and E are the coded values for the liquid–solid ratio, ultrasonic time, ultrasonic temperature, microwave power, and microwave time, respectively.

Similar to EAE, the parameters were also evaluated; it was confirmed that this model can be used to analyze and predict the effects of factors on the extraction rate in UMAE, as shown in Table S2.

##### Response Surface Analysis of UMAE

As shown in Figure S1F,G, at a higher level of liquid–solid ratio, the polysaccharides obtained from extraction increased first and then decreased with the increase in ultrasonic time. Similarly, Figure S1J,K,N–Q,X,Y indicate highly significant interactions of AD, BC, BD, and DE, respectively, and Figure S1T,U indicate significant interactions of CD. Figure S1H,I,L,M,R,S,V,W indicate that AC, AE, BE, and CE interacted insignificantly.

##### Verification of the Predictive Model of UMAE

The optimum UMAE parameters are a liquid–solid ratio of 30:1 (mL/g), an ultrasonic time of 96.9083 min, an ultrasonic temperature of 50.6721 °C, microwave power of 337.9950 W, and microwave time of 3.3927 min, and the expected maximum yield was 12.5179%. Considering the simplicity of the extraction process, the extraction parameters were slightly adjusted: a liquid–solid ratio of 30:1 (mL/g), an ultrasonic time of 100 min, an ultrasonic temperature of 50 °C, microwave power of 340 W, and a microwave time of 3.5 min. According to the optimal extraction conditions, the actual yield of polysaccharides was 12.67 ± 0.31%. The RSM model was validated by this experimental result, which showed that this model was suitable for the UMAE process.

### 2.3. Optimization of Hot Water Extraction (HWE) of Polysaccharides from D. huoshanense

Hot water extraction is a convenient and commonly used method for extracting polysaccharides. And the extraction efficacy is affected by the liquid–solid ratio, extraction temperature, and extraction time. The optimization results are as follows:

#### 2.3.1. Optimization Results of HWE with the Single-Factor Test

The optimization results of HWE with a single-factor test are shown in Figure S2. As shown in Figure S2A, similar to EAE, the extraction yield reached a maximum at a liquid–solid ratio of 40:1 and reached a maximum value of 14.30%. And it is clear from Figure S2B that the yield of polysaccharides rises with increasing temperature, from 60 to 100 °C. From Figure S2C, it can be seen that the yield of polysaccharides was significantly affected when the extraction time was increased to 120 min. Thus, the factors, including the liquid–solid ratio (30:1, 40:1, 50:1 (mL/g)), extraction temperature (60, 80, 100 °C), and extraction time (60, 120, 180 min), were optimized further with BBD compared with the other two extraction methods. The experimental design for the independent variables is shown in Table S3.

#### 2.3.2. Optimization Results of HWE with RSM with Box–Behnken Design

##### Model Fitting and Statistical Analysis of HWE

In HWE, the second-order polynomial equation is shown below:

Y = 14.31 + 0.71 × A − 0.8787 × B + 1.96 × C + 0.315 × AB + 0 × AC − 1.74 × BC − 1.45 × A^2^ − 1.23 × B^2^ − 0.3113 × C^2^ (3)

where Y is the yield of polysaccharides and A, B, and C are the coded values for the extraction temperature, extraction time, and liquid–solid ratio, respectively.

Similar to UMAE, the model can be used to predict and analyze the influence of various extraction conditions on the yield of DHP-H in an extraction test (Table S4).

##### Response Surface Analysis of HWE

As shown in Figure S2H,I, the yield of polysaccharides in DHP showed an increasing trend with the increase in the liquid–solid ratio at a relatively low extraction time. Moreover, the result indicates a highly significant interaction between the liquid–solid ratio and extraction time. However, Figure S2D–G shows the insignificant interactions of AB and AC.

##### Verification of the Predictive Model of HWE

The optimum HWE parameters are an extraction temperature of 82.7159 °C, an extraction time of 60.0008 min, and a liquid–solid ratio of 50:1 (mL/g), and the expected maximum yield was 17.3769%. Considering the simplicity of operation during the extraction process, the extraction parameters were slightly adjusted: an extraction temperature of 85 °C, an extraction time of 60 min, and a liquid–solid ratio of 50:1 (mL/g). Based on the optimum conditions, a high yield of polysaccharides of 17.54 ± 0.31% was obtained from practical experiments. The RSM model has been validated with the experimental results.

### 2.4. Chemical Characteristics of Polysaccharides Extracted with Different Methods

Detailed investigations were carried out to examine the chemical characteristics of DHP prepared with various extraction techniques. Ten extractions based on the optimized conditions were performed for each extraction method to collect polysaccharides for further structure characterization and activity evaluation. The average yield of 10 extractions with EAE, UMAE, and HWE was 14.30 ± 0.25%, 12.61 ± 0.06%, and 16.16 ± 0.31% (Table 3). And the coefficient of variation (C.V.) of average yield with the confirmation experiment is 0.42% for EAE, 0.48% for UMAE, and 8.5% for HWE. The C.V. of HWE is relatively high, which is probably because the extraction time of HWE is relatively long. During the extraction process, the sample easily sediments at the bottom, resulting in insufficient mixing with the extraction solvent, ultimately affecting the extraction efficiency. The results in Table 3 clearly show that the traditional HWE had the highest extraction yields. In DHP-E, DHP-UM, and DHP-H, the carbohydrate content is 68.91 ± 0.32%, 71.19 ± 0.13%, and 75.32 ± 0.02%; the uronic acid content is 4.18 ± 0.23%, 3.52 ± 0.11%, and 4.80 ± 0.26%; and the protein content is 13.08 ± 0.18%, 8.28 ± 0.21%, and 7.17 ± 0.23%, respectively. DHP-E showed a high content of protein compared with DHP-UM and DHP-H, which is probably caused by the residual enzymes, even though the heating denaturation was performed. Although most phenolic compounds were removed with ethanol extraction and precipitation, a few phenolic compounds were also detected in DHPs. The contents of total phenolic compounds in DHPs ranged from 1.56 ± 0.41 to 4.20 ± 0.23 mg GAE/g, which probably contributes to the UV absorption. The results confirm that the extracts mainly consist of polysaccharides. Hence, it can be concluded that all three extracts share similar chemical properties. However, the detailed structure information related to bioactivity needs further verification.

### 2.5. Analysis of UV and FT-IR

**Figure 2:**
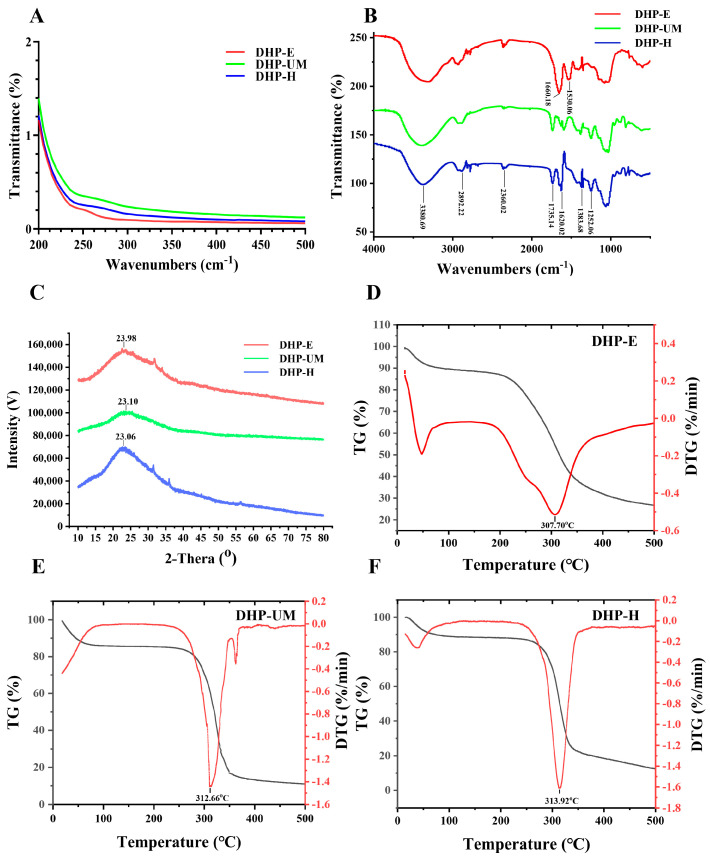
(**A**) UV spectrum, (**B**) FT-IR spectrum, and (**C**) XRD of DHP-E, DHP-UM, and DHP-H. TG and DTG curve of DHP-E (**D**), DHP-UM (**E**), and DHP-H (**F**). The black line is the TG curve, and the red line is the DTG curve.

The physicochemical properties are summarized in Figure 2. As shown in Figure 2A, DHP-H, DHP-E, and DHP-UM showed small absorption peaks at 260 nm to 280 nm, indicating a small number of phenolic compounds or proteins in DHPs. This result is confirmed by the protein and total phenolics content data shown in Table 3.

FT-IR spectroscopy was also utilized to estimate the structural characteristics of DHPs. The FT-IR spectra of DHP-E, DHP-UM, and DHP-H are displayed in Figure 2B. The characteristic stretching absorption peaks were observed at 3380.69 cm^−1^ and 2892.22 cm^−1^, corresponding to the absorption of O-H and C-H, which indicate -OH and the skeleton of polysaccharides, respectively [21]. The signal at 1735.14 cm^−1^ was the esterified carboxylic groups, which indicates the methyl esterization of carboxylic groups in acidic polysaccharides [21]. Furthermore, the signal at 1620.02 cm^−1^ indicated the presence of C=O, indicating that the DHPs were acidic polysaccharides [21]. The signal at 1383.68 cm^−1^ was related to C-H or O-H [22]. Furthermore, the signal at 1252.06 cm^−1^ was the C-O-C, consisting of the existence of -OCH_3_ and corresponding to esterified carboxylic groups in acidic polysaccharides [23]. The peaks in the absorbance range of 1200–1000 cm^−1^ indicate the presence of stretching vibrations associated with C-O-C, C-O-H, and C-C, suggesting the existence of the pyranose structure [23]. It is worth noticing that the signals at 1660.18 cm^−1^ and 1530.06 cm^−1^ in DHP-E indicated that there is a relatively high protein content, which corresponds to the results of chemical analysis [21].

### 2.6. X-ray Diffraction Analysis and Thermal Analysis

X-ray diffraction (XRD) is an essential technique for examining polymer crystal structures, enabling a more comprehensive examination of polysaccharide structures [24]. Typically, crystalline materials exhibit sharp narrow diffraction peaks, whereas amorphous components display broad peaks [25]. The X-ray diffraction (XRD) results of the DHPs are shown in Figure 2C. In the range of 10° to 80°, the diffraction patterns at 2θ exhibited similar peak shapes, and both broad and weak diffraction peaks were observed at ~23°, indicating low overall crystallinity and the presence of amorphous interiors of DHPs. The width and intensity of diffraction peaks in DHP-E and DHP-UM were smaller than those of DHP-H, indicating that the crystalline structure of DHPs is influenced by structures such as molecular weight and glycosidic linkages, as reported in a previous study [7]. These structural properties are critical because they have a direct impact on the stability, chemical resistance, and solubility of the polysaccharides [26].

The quantitative measurement of material mass changes due to dehydration, decomposition, and oxidation can be achieved by utilizing the thermal stability of biomolecules [27,28]. The combined TG-DTG method was employed to determine the thermal properties in this experiment (Figure 2D–F). As presented in Figure 2D, the thermal degradation of DHP-E occurs over a broader temperature range, and this is probably caused by the high content of protein in DHP-E [28]. The DTG (red line) of DHP-UM (Figure 2E) exhibited three weight loss stages, while DHP-H (Figure 2F) exhibited two weight loss stages. The pyrolytic curves of DHPs tended to be similar in the first stage of weight loss (15.05–120 °C), which might be attributed to the evaporation of the free water and binding water to the polysaccharides [27,28]. During the second stage, characterized by temperatures in the ranges of 182.97–370.69 °C (DHP-E), 234.55–353.67 °C (DHP-UM), and 233.70–377.55 °C (DHP-H), there was a significant and pronounced weight reduction. When the temperature rose to 307.70 °C (DHP-E), 312.66 °C (DHP-UM), and 313.92 °C (DHP-H), a significant decrease in the weight of polysaccharides was observed, potentially due to the depolymerization and decomposition of polysaccharides [27,28] In addition, during the third phase between 353.67 and 370.25 °C (DHP-UM), there was a reduction in weight, potentially caused by the decomposition of other macromolecules [29]. Consequently, these results showed that DHPs had relative thermal stability below 313.92 °C. It is worth mentioning that the thermal stability of DHP-E and DHP-UM is weaker than that of DHP-H.

### 2.7. Scanning Electron Microscopy (SEM) Analysis

**Figure 3:**
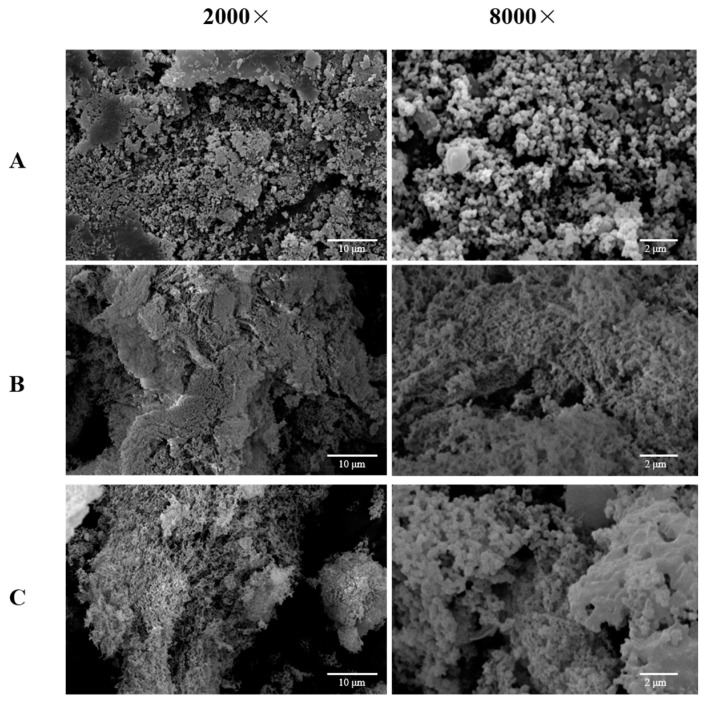
SEM images of DHP-E (**A**), DHP-UM (**B**), and DHP-H (**C**): 2000× (**left**), 8000× (**right**).

The biological function of a polysaccharide is influenced by its stereo-shape. Microstructural images of the *D. huoshanense* treated with EAE, UMAE, and HWE are displayed in Figure 3. The microstructural characteristics of the polysaccharides obtained varied among the three extraction methods. DHP-E possessed a loose surface and was composed of many small particles, which suggested that cellulase could catalyze and hydrolyze the cell wall of the *D. huoshanense* to promote the polysaccharides dissolving into the solvent and could also decompose the polysaccharides to small ones (Figure 3A). In addition, DHP-UM was sponge-like, and DHP-H was more compact than DHP-UM. Ultrasonic-assisted extraction mainly depends on the cavitation effect that can split the *D. huoshanense* cell walls and can also change the surface looseness of polysaccharides (Figure 3B,C). DHP-H had a massive and rough surface with many irregular bulges with inconsistent apertures (Figure 3C). According to reports, extraction techniques may affect the branched structures and crosslinking networks in *Bletilla striata* polysaccharides, thus endowing different surface topographies and appearances [7]. The dissimilarities observed in the three DHPs’ microstructures imply that the different extraction methods had disparate impacts on the surface morphology of the macromolecules, which might have endowed the polysaccharides with unique physical characteristics.

### 2.8. Analysis of Molecular Weights and Monosaccharide Compositions

**Figure 4:**
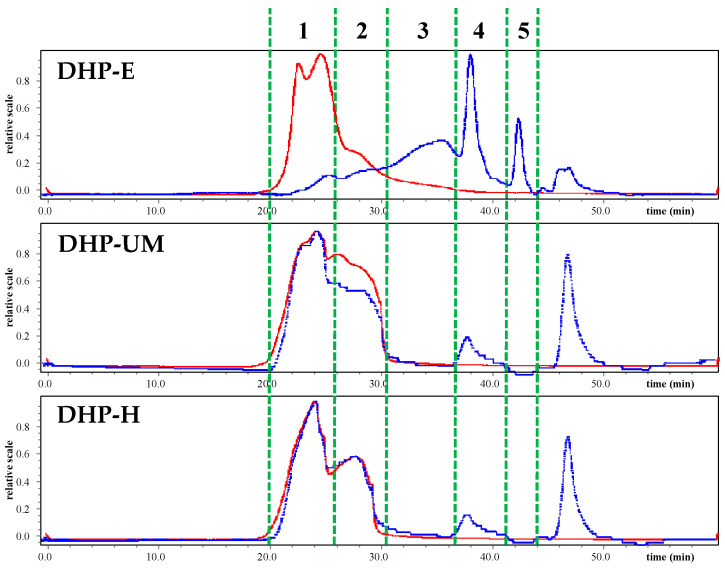
HPSEC-MALLS-RID chromatogram of DHPs for molecular weight determination (red line: light scattering (LS); blue line: refractive index (RI)). The chromatograms of the different extraction methods are divided into five sections labeled by green dashed lines.

The biological activity of polysaccharides is usually related to their molecular weight distribution and constituent monosaccharides [30,31]. Figure 4 is the chromatogram of HPSEC-MALLS-RID, and it can be seen that the three polysaccharides extracted with different methods had obviously different molecular weight distributions. The Mw of DHP-E, DHP-UM, and DHP-H is summarized in Table 4. The fractions at peak 1 and peak 2 have a large molecular weight as the signal of MALLS is high (red line), and these components are also present in large amounts in DHP-UM and DHP-H, as indicated by the strong signal of RI (blue line). However, the contents of these large-molecular-weight fractions are low in DHP-E compared with DHP-UM and DHP-H. Furthermore, the fractions with Mw 2.12 × 10^4^ and 5.23 × 10^4^ Da only existed in DHP-E and in a large amount. The Mw of the fraction at peak 4 in DHP-E, DHP-UM, and DHP-H was 2.63 × 10^4^, 7.20 × 10^4^, and 4.98 × 10^4^, and this fraction is present in a relatively large amount in DHP-E. The peak that appeared after 45 min is the small-molecule compounds. The result of Mw analysis showed that polysaccharides with higher molecular weight detected in DHP-H and DHP-UM did not appear in DHP-E, which indicates that the degradation of polysaccharides caused by enzymes changed the molecular weight distribution of polysaccharides and probably finally affected their bioactivities [32]. The previous study also showed that different extraction methods have a significant impact on the molecular weight of polysaccharides. *Morinda citrifolia* L. (Noni) polysaccharides (NPs) extracted based on HWE, pulsed electric field-assisted extraction (PEFAE), and UAE have been reported to affect the molecular weight, whereas UAE-NP had the smallest molecular weight [33].

**Figure 5:**
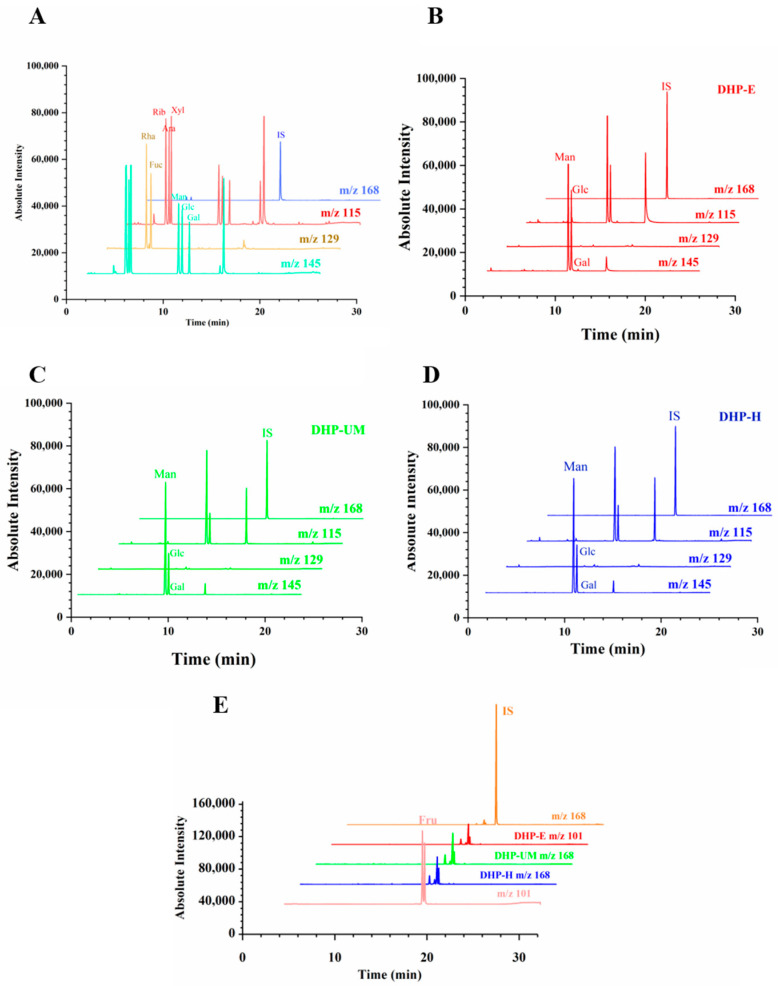
Extracted ion GC-MS chromatograms of (**A**) aldose mixed standard containing mannose (Man), glucose (Glc), galactose (Gal), rhamnose (Rha), fucose (Fuc), ribose (Rib), arabinose (Ara), xylose (Xyl), and internal standard (IS). DHP-E (**B**), DHP-UM (**C**), and DHP-H (**D**). Typical extracted ion chromatograms of fructose (Fru) standard, DHP-E, DHP-UM, and DHP-H (**E**).

The amounts of individual monosaccharides in DHPs were assessed using GC-MS in this study. Figure 5 shows the extracted ion GC-MS chromatograms of different ions. Figure 5A is the chromatogram of mixed standards of aldose. Figure 5B–D presents the samples of DHP-E, DHP-UM, and DHP-H. Figure 5E is the chromatogram of the standards and samples of ketose. *m*/*z* 145 is the main characterized fragments of Man, Glc, and Gal, thereby used for the calculation of the molar ratio. Simultaneously, *m*/*z* 129 for Rha and Fuc, *m*/*z* 115 for Rib, Xyl, and Ara, *m*/*z* 101 for Fru, and *m*/*z* 168 for internal standard (IS) were used for the calculation of the molar ratio. The monosaccharide compositions are shown in Table 4, and it can be seen that varying molar ratios of Glc and Man are present in DHP-E (68.49 ± 1.13% and 31.51 ± 1.13%), DHP-UM (86.03 ± 0.81% and 13.97 ± 0.81%), and DHP-H (85.22 ± 0.45% and 14.78 ± 0.45%). And only a small amount of Gal was detected. According to the findings, DHP primarily consisted of Glc and Man. These results are consistent with those of previous studies on *D. huoshanense* [17]. The content of Glc is relatively high in DHP-E. In summary, EAE and UMAE treatments resulted in the hydrolytic cleavage of polysaccharide chains and the breakage of intermolecular hydrogen bonds, consequently impacting the composition of monosaccharides [21,34,35,36]. The results indicated that the extraction techniques had an impact on the monosaccharide composition of the DHP samples, while the monosaccharide varieties remained unaltered.

### 2.9. Methylation Analysis

**Figure 6:**
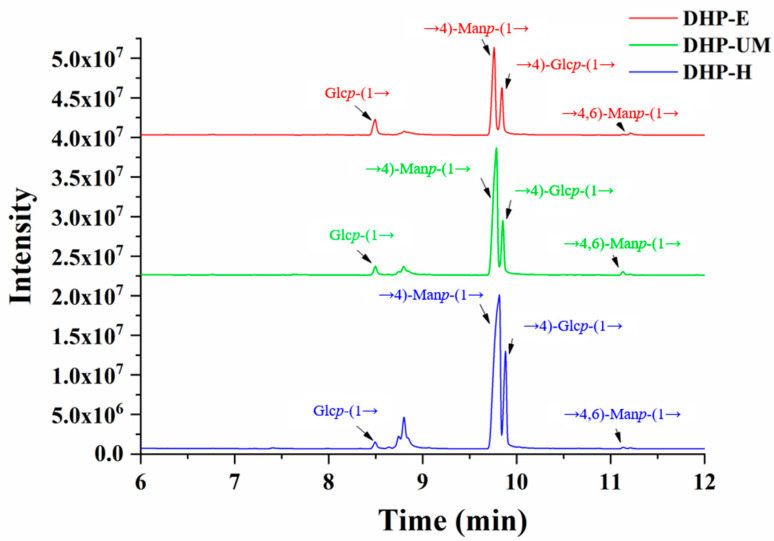
GC-MS total ion chromatography of partially methylated alditol acetate of DHP-E, DHP-UM, and DHP-H.

In addition, methylation analysis was performed with GC-MS analysis of partially methylated alditol acetate, and the results are shown in Figure 6 and Table 5. The results showed that DHP-E, DHP-UM, and DHP-H all contained four types of residues, including Glc*p*-(1→, →4)-Man*p*-(1→, →4)-Glc*p*-(1→, and →4,6)-Man*p*-(1→; their molar ratios were 1.00: 4.63: 2.51: 0.14; 1.00: 16.19: 5.37: 0.31; and 1.00: 36.03: 12.57: 0.18, respectively. The results of glycosidic bond types and ratios in DHPs are also summarized in Table 5. It can be seen that the glucomannan structure consisting of →4)-Glc*p*-(1→ and →4)-Man*p*-(1→ as the backbone is the major polysaccharide in *D. huoshanense*, which is consistent with the structure of separated homogeneous polysaccharides from *D. huoshanense* [2]. The higher ratios of →4)-Glc*p*-(1→ and →4)-Man*p*-(1→ in DHP-UM (5.37:16.19) and DHP-H (12.57:36.03) indicated the existence of relatively longer backbone structures, which is consistent with the higher Mw distribution of polysaccharides contained in DHP-UM and DHP-H. The higher percentage ratio of →4,6)-Man*p*-(1→ in DHP-E (1.69%) compared with DHP-UM (1.36%) and DHP-H (0.36%) also indicated that more branch structures may exist in DHP-E and probably contributed to the enhancement in activity [4].

### 2.10. Antioxidant Activity of DHPs

#### 2.10.1. DPPH Radical Scavenging Activity of DHPs

**Figure 7:**
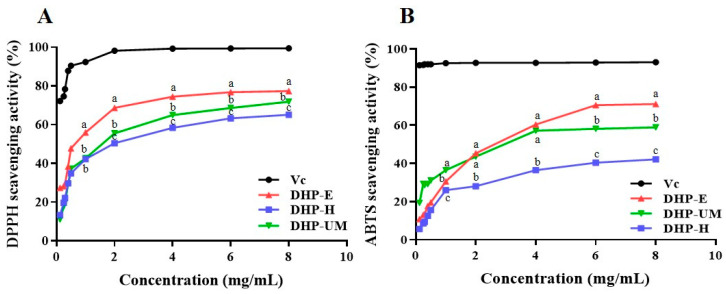
Antioxidant activity of DHP-E, DHP-UM, and DHP-H. DPPH radical scavenging activity (**A**) and ABTS+ radical scavenging activity (**B**). Vitamin C (V_C_) is used for the positive control. Significant (*p* < 0.05) differences are shown by data bearing different letters (a–c).

DPPH radical scavenging relies on the hydrogen-donating ability of antioxidants and has gained widespread recognition as a valuable technique for estimating the free radical scavenging activities of antioxidants [35]. The results of the DPPH radical scavenging activity of DHP samples are showcased in Figure 7A. All DHP samples demonstrated the ability to scavenge DPPH, and the scavenging capacity displayed a concentration-dependent approach with concentrations ranging from 0.125 to 8.0 mg/mL. DHP-E showed stronger DPPH radical scavenging activity than DHP-UM and DHP-H. However, it was still lower than the positive control group. Therefore, these results indicated that DHP-E, DHP-UM, and DHP-H can donate hydrogen to scavenge DPPH radicals.

#### 2.10.2. ABTS Radical Scavenging Activity of DHPs

The ABTS radical scavenging method has gained widespread acceptance as a valuable method for assessing the overall antioxidant capacity of natural substances, and compared with DPPH, it is more suitable for antioxidant evaluation for water-soluble components [36]. The consequences of ABTS radical scavenging are depicted in Figure 7B. The results demonstrated that all DHP samples exhibited noticeable effectiveness in ABTS radical scavenging, and concentrations ranging from 0.125 to 8.0 mg/mL showed a concentration-dependent scavenging ability. The ABTS scavenging activities of all DHP samples varied and followed the descending order of DHP-E > DHP-UM > DHP-H. The findings demonstrated that DHP-E had a better ABTS radical scavenging capacity than the other DHP samples, but it was weaker than ascorbic acid in each concentration. DHPs exhibited good antioxidant activity in both these two systems.

Differences in the antioxidant activity of polysaccharides extracted from the same source arise from variations in molecular weight, monosaccharide compositions, and glycosidic bonds [30]. Previous studies have reported that polysaccharides with a lower molecular weight usually have more free hydroxyl groups at the same mass concentration, thus exerting stronger antioxidant capacity [31]. Therefore, low-molecular-weight DHP-E favors the exposure of the active site, which contributed to the antioxidant activity. Meanwhile, more branching structures also facilitate the exposure of active groups in polysaccharides. The relatively high ratio of →4,6)-Man*p*-(1→ in DHP-E and DHP-UM compared with DHP-H (Table 5) also contributed to their antioxidant activity. Simultaneously, the other components contained in DHPs, such as protein and phenolic components, also contributed to their bioactivity, which needs further purification to confirm.

## 3. Materials and Methods

### 3.1. Materials and Chemicals

*Dendrobium huoshanense* C. Z. Tang et S. J. Cheng was collected from the Dendrobium planting base of West Anhui University (Lu’an, Anhui, China). Monosaccharide reference substances inducing mannose (Man), fucose (Fuc), galactose (Gal), glucose (Glc), ribose (Rib), rhamnose (Rha), arabinose (Ara), xylose (Xyl), and fructose (Fru) were purchased from Sigma-Aldrich (St. Louis, MO, USA). 1,1-diphenyl-2-picryl-hydrazyl (DPPH), 2,2-azino-bis (3-ethylbenzothiazoline-6-sulphonic acid) (ABTS), and trifluoroacetic acid (TFA) (HPLC grade) were purchased from Maclin Biochemical Co., Ltd. (Shanghai, China). Cellulase was purchased from Shanghai Yuanye Bio-Technology Co., Ltd. (Shanghai, China). Other reagents used in this study were purchased from Macklin Biochemical Co., Ltd. (Shanghai, China).

### 3.2. Optimization of the Extraction Method with a Single-Factor Test

The samples were ground into fine powder. After extraction with 60% ethanol in a Soxhlet apparatus for 2 h to remove the small-molecule constituents and oligosaccharides, the samples were dried for subsequent extraction [36].

Enzyme-assisted extraction (EAE): The pretreated powder was then used for the extraction of polysaccharides using the enzyme-assisted method [37]. In total, 0.5 g powder was weighed, and different volumes of distilled water were added to form the liquid-to-solid ratios of 20:1, 30:1, 40:1, and 50:1 (*v*/*w*). The supernatant was adjusted to pH 4.5, 5.0, and 5.5 with citric acid buffer (1%), and the relevant 2% cellulase enzyme preparations were added. The sample was placed in a thermostatic water bath at temperatures of 40, 55, and 70 °C for different extraction times of 30, 60, 90, and 120 min. After enzymatic hydrolysis, the enzyme was inactivated in hot water at 90 °C for 10 min.

Ultrasound–microwave-assisted extraction (UMAE): In total, 0.5 g powder was weighed, and distilled water (20:1, 30:1, 40:1, v/w) was added. The mixture was extracted in a microwave coupled with an ultrasonic extractor (Scientz-IIDM, Scientz Biotechnology Co., Ltd. Ningbo, China) under ultrasonic conditions for 30, 60, 90, and 120 min at temperatures of 20, 40, and 60 °C using an ultrasound power of 105 W. The mixture was then extracted at microwave power levels of 200, 400, and 600 W, with microwave extraction times of 3, 4, and 5 min.

Hot water extraction (HWE): The pretreated powder was then used for the extraction of polysaccharides with the hot water method using a constant-temperature heating magnetic stirrer (DF-101S, Shanghai Lingke Industrial Development Co., Ltd. Shanghai, China) with a condenser at liquid–solid ratios of 20:1, 30:1, 40:1, and 50:1, extraction temperatures of 60, 80, and 100 °C, and extraction times of 60, 120, and 180 min.

All extractions were carried out under magnetic stirring. After extraction, an appropriate amount of water was added to the extract to replenish the weight lost during extraction. After extraction, the extracts were centrifuged for 10 min at 5000 rpm. The resulting supernatant was concentrated, and 95% ethanol was added with continuous stirring to achieve a final concentration of 80% ethanol. This solution was stored for 24 h at 4 °C. The mixture was then centrifuged at 5000 rpm for 10 min to collect the precipitated polysaccharides. The polysaccharide content was measured using the phenol sulfate method. The yield of polysaccharides was calculated according to Equation (4).

(4) $$Y\;(\%)=\frac{C\;\times \;V\times \;n}{W}\times 100$$

Y is the yield of polysaccharide; V is the total volume of extraction solution, mL; n is the dilution factor; if the concentration of the extraction solution is too high and exceeds the quantitative range, it should be diluted; W is the weight of material, g; and C is the concentration of polysaccharide, mg/mL.

### 3.3. Optimization of the Extraction Method with BBD

There are complex interactions between parameters in multiple extraction methods. RSM can fit the relationship between multiple variables and response values through equations to obtain the optimal combination of parameters. The operational parameters involved in the EAE, UMAE, and HWE processes were designed using the Box–Behnken design method based on a one-way test. The appropriateness of the model equations was expressed by the coefficient of determination R^2^, and regression coefficient significance and statistical significance were tested using the F-test, with *p* < 0.05 indicating significance [15].

### 3.4. Characterization of Polysaccharides from D. huoshanense Extracted with Different Methods

#### 3.4.1. Chemical Analysis

Total sugar content was analyzed using the phenol-sulfuric acid method [38]. Total uronic content was determined using carbazole-sulfuric acid with galacturonic acid as the standard [39]. Nitrogen concentration was determined with the Dumas combustion N determination method on an elemental analyzer (Thermo Fisher, FLASH 2000, Waltham, MA, USA), following wrapping 1.0 mg of the powder in a crucible. Subsequently, a 6.25 nitrogen-to-protein conversion factor was used to calculate the content of protein [40]. The Folin–Ciocalteu assay was used to determine the total phenolics [41].

#### 3.4.2. Molecular Weight Analysis

The molecular weight of DHPs was tested using high-performance size-exclusion chromatography–multi-angle laser light scattering–refractive index detector (HPSEC-MALLS-RID). The HPSEC-MALLS-RID system was composed of a pump (LC-40D XS, Shimadzu, Kyoto, Japan), SB-806HQ (8.0 mm × 300 mm) and SB-804HQ (8.0 mm × 300 mm, Showa Denko KK, Tokyo, Japan) columns connected in series, a MALLS detector (Wyatt Technology, Santa Barbara, CA, USA), and an RID (RefractoMax 520, ERC, Shimadzu, Kyoto, Japan). Briefly, the polysaccharide samples were dissolved in 0.9% aqueous NaCl solution at a concentration of 7.0 mg/mL and filtered through 0.22 μm membranes for analysis at 30 °C, using a mobile phase of a 0.9% aqueous NaCl at a 0.5 mL/min flow rate. The injection volume was 90 µL. A dn/dc value of 0.1380 (mL/g) was applied to determine the molar mass. Data were imported into Astra software (version 5.3.4, Wyatt Tech. Corp. Santa Barbara, CA, USA) for analysis.

#### 3.4.3. Monosaccharide Analysis

Briefly, four parallel DHPs (each 3.0 mg) were hydrolyzed with 2 M TFA (1 mL) for 8 h at 95 °C. For ketoses, two parallel hydrolysates were dissolved in 0.5 mL pyridine, and 10 mg of methoxyamine hydrochloride was added; the sample was incubated at 70 °C for 60 min. Then, 0.5 mL of acetic anhydride was added, and the sample was incubated for 60 min at 45 °C. For aldoses, two parallel hydrolysates were dissolved in 0.5 mL pyridine, and 10 mg hydroxylamine hydrochloride was added; then, the sample was incubated for 30 min at 90 °C. Finally, acetic anhydride (0.5 mL) was added and incubated for 30 min at 90 °C. After drying with a nitrogen evaporator, the resulting product was dissolved in 1 mL methanol, which contained 100 μg myo-inositol hexaacetate as internal standard. The monosaccharide derivatization products were determined with GC-MS (Shimadzu QP2020 NX) using HP-5MS (30 m × 0.25 mm ID, 0.25 μm film thickness, Agilent, Santa Clara, CA, USA) [42].

#### 3.4.4. Methylation Analysis

DHP (3.0 mg) was dissolved in 1.0 mL of dimethyl sulfoxide (DMSO) and then combined with NaOH (10 mg) and ultrasound-incubated for 20 min. Afterwards, CH_3_I was added under ice-bath conditions and ultrasound-incubated for 30 min, and the above steps were repeated two times. The partially methylated DHP was hydrolyzed for 30 min with 0.5 M TFA and then dried. Following a 2-h reduction with NaBH_4_, the monosaccharide underwent subsequent treatment with acetic anhydride and pyridine for 30 min to convert the released monosaccharides to partially methylated alditol acetate, which was finally analyzed with GC-MS [42].

#### 3.4.5. Fourier-Transform Infrared Spectroscopy (FT-IR) and UV Spectroscopy

For IR spectroscopy, DHP was mixed with KBr powder, ground and pressed, and analyzed using an FT-IR spectrometer (Thermo Fisher, Nicolet iS5, Waltham, MA, USA) in the range of 500 to 4000 cm^−1^.

For the UV spectrum, the DHP was prepared into 1.0 mg/mL with purified water, and the UV full-wavelength scanning was carried out at 200–500 nm with universal analysis on a UV-Vis spectrophotometer (TU-1901, Beijing Purkinje General Instrument Co., Ltd., Beijing, China).

#### 3.4.6. Thermal Analysis and X-ray Diffraction (XRD) Analysis

Thermogravimetric analysis (TGA) of DHP was conducted using a Netzsch thermogravimetric analyzer (Netzsch STA449 F3 Jupiter, Selb, Germany). DHP was filled into an alumina crucible and then heated to 500 °C [42]. Thermogravimetric characteristics of DHP (1.5 mg in a platinum crucible) were determined with a thermal analyzer heated at a rate of 10 ◦C/min in the range of 30 °C to 500 °C under a nitrogen atmosphere flowing at 100 mL/min.

The lyophilized powder of DHP was analyzed with an X-ray diffractometer (Ultima IV, Rigaku, Akishima-shi, Tokyo, Japan). Acquisitions were made in the 2θ range from 10 to 80°.

#### 3.4.7. Scanning Electron Microscopy (SEM)

The surface characteristics of polysaccharides were examined using an SEM model (JSM-7500F, JEOL Ltd., Akishima-shi, Tokyo, Japan). The SEM was operated at an acceleration voltage of 5 kV to investigate the surface morphology [42]. Before being observed, the samples underwent a gold sputtering process, which occurred in a reduced-pressure environment.

### 3.5. Assay for Antioxidant Activity

#### 3.5.1. DPPH Radical Scavenging Activity

DPPH (0.2 mM, ethanol, 2.0 mL) with DHP (2.0 mL) solution at different concentrations (0.125–8 mg/mL) was prepared at 25 °C and then further reacted for 30 min in the dark before centrifugation for 10 min at 10,000 rpm and absorbance at 517 nm to measure the absorbance of the mixture. The DPPH free radical scavenging activity was calculated by using Equation (5):

(5) $$\mathrm{DPPH}\;\mathrm{scavenging}\;\mathrm{rate}\;(\%)=(1-\frac{Ai-Aj}{A0})\times 100$$

where A_0_ is the absorbance of the solution including 2 mL of DPPH and 2 mL of ethanol; A_i_ is the absorbance of the sample reaction solution; and A_j_ is the absorbance of the solution including 2 mL of sample and 2 mL of ethanol.

#### 3.5.2. ABTS Radical Scavenging Activity

The ABTS radical scavenging activity of the polysaccharides was evaluated according to a similar method reported previously [36]. ABTS solution was obtained by adding K_2_S_2_O_8_ (2.45 mmol/L) into ABTS (7 mmol/L) aqueous solution, which was reacted for 12–16 h at 25 °C in the dark. Then, the resulting mixture was further diluted with phosphate buffer (pH = 7.4) to achieve an absorbance of 0.70 ± 0.02 at 734 nm. Aqueous solutions containing different polysaccharides (5 mL, 0.125–8 mg/mL) were added to the above diluted solution containing ABTS (0.2 mL) and mixed thoroughly. The solution was allowed to stand at 25 °C for 60 min, and then the absorbance at 734 nm was measured. The free radical scavenging capacity of ABTS was calculated according to the same principle of Equation (5).

### 3.6. Statistical Analyses

All experiments were conducted in duplicate, and the results were presented as mean ± standard deviation (SD). Design Expert 11.0 software (Stat-Ease lNC, Scottsdale, IL, USA) was used to design BBD experiments for RSM and statistical analysis. To determine the significance of the model, a one-way analysis of variance (ANOVA) was performed. The goodness of fit of the polynomial model equation was evaluated using the coefficient of determination (R^2^). Additionally, the significance of the regression coefficients was assessed through an F-test; *p* < 0.05 means significant. Duncan’s multiple range test (using SPSS software 22) was used to analyze the statistical significance of differences between the groups in the signal factor test and antioxidant test. A significance difference was judged to exist at a level of *p* < 0.05.

## 4. Conclusions

In this study, three different extraction methods were optimized and used to obtain the polysaccharides from *D. huoshanense*. The results showed that HWE exhibited the best extraction efficacy. The pH, temperature, and liquid–solid ratio showed a significant interaction for EAE, and the ultrasonic time, temperature, microwave power, and liquid–solid ratio showed a significant interaction for UMAE. The three obtained DHPs showed similar characteristics in crystal. DHP-E showed a different thermal stability to DHP-UM and DHP-H, which display a broader temperature range during thermal degradation. DHP-E also displayed irregular granularity and a very rough surface compared with DHP-UM and DHP-H in surface morphology. Polysaccharides with a higher molecular weight detected in DHP-H and DHP-UM did not appear in DHP-E due to enzymatic degradation. The content of Glc is relatively high in DHP-E. Four glycosidic bond types, including Glc*p*-(1→, →4)-Man*p*-(1→, →4)-Glc*p*-(1→, and →4,6)-Man*p*-(1→, were found in *D. huoshanense*. The different ratios of glycosidic bond types indicated that more branch structures probably exist in DHP-E, which may be beneficial for the enhancement in activity. And in the following activity test, DHP-E indeed exhibited strong free radical scavenging ability (DPPH and ABTS), which was attributed to the lower molecular weight and the branched structure. However, the structural analysis of DHP and the mechanism that promotes the exertion of bioactivities have not been thoroughly investigated. This study provides data support for further effective utilization of *D. huoshanense*, which is a scarce resource but has definite therapeutic effects.

## Figures and Tables

**Table 1:** Experimental design for independent variables and the response of the extraction yield of EAE in Box–Behnken design.

Run	Coded Variables				Uncoded Variable				Response
	A	B	C	D	A (Extraction Time, min)	B (Liquid–Solid Ratio, (mL/g))	C (Extraction Temperature, °C)	D (Extraction pH)	Y (the Extraction Yield of DHP %)
1	−1	−1	0	0	30	30:1	55	5	12.56
2	1	−1	0	0	90	30:1	55	5	11.13
3	−1	1	0	0	30	50:1	55	5	12.54
4	1	1	0	0	90	50:1	55	5	12.29
5	0	0	−1	−1	60	40:1	40	4.5	11.30
6	0	0	1	−1	60	40:1	70	4.5	12.68
7	0	0	−1	1	60	40:1	40	5.5	13.52
8	0	0	1	1	60	40:1	70	5.5	11.03
9	−1	0	0	−1	30	40:1	55	4.5	12.94
10	1	0	0	−1	90	40:1	55	4.5	12.49
11	−1	0	0	1	30	40:1	55	5.5	11.52
12	1	0	0	1	90	40:1	55	5.5	12.58
13	0	−1	−1	0	60	30:1	40	5	11.51
14	0	1	−1	0	60	50:1	40	5	11.74
15	0	−1	1	0	60	30:1	70	5	10.70
16	0	1	1	0	60	50:1	70	5	11.98
17	−1	0	−1	0	30	40:1	40	5	12.19
18	1	0	−1	0	90	40:1	40	5	12.62
19	−1	0	1	0	30	40:1	70	5	11.35
20	1	0	1	0	90	40:1	70	5	12.03
21	0	−1	0	−1	60	30:1	55	4.5	10.93
22	0	1	0	−1	60	50:1	55	4.5	14.02
23	0	−1	0	1	60	30:1	55	5.5	12.46
24	0	1	0	1	60	50:1	55	5.5	11.05
25	0	0	0	0	60	40:1	55	5	13.98
26	0	0	0	0	60	40:1	55	5	14.75
27	0	0	0	0	60	40:1	55	5	14.51
28	0	0	0	0	60	40:1	55	5	14.70
29	0	0	0	0	60	40:1	55	5	14.47

**Table 2:** ANOVA for response surface quadratic model for the yield of EAE.

Source	Sum of Squares	Df	Mean Square	F-Value	*p*-Value	
Model	38.92	14	2.78	16.08	<0.0001	Significant
A-Extraction time	0.0001	1	0.0001	0.0008	0.9782	
B-Liquid–solid ratio	1.56	1	1.56	9.04	0.0094	
C-Extraction temperature	0.806	1	0.806	4.66	0.0487	
D-Extraction pH	0.4033	1	0.4033	2.33	0.149	
AB	0.3481	1	0.3481	2.01	0.1778	
AC	0.0156	1	0.0156	0.0904	0.7681	
AD	0.57	1	0.57	3.3	0.0909	
BC	0.2756	1	0.2756	1.59	0.2274	
BD	5.06	1	5.06	29.28	<0.0001	***
CD	3.74	1	3.74	21.65	0.0004	***
A^2^	6.61	1	6.61	38.21	<0.0001	***
B^2^	13.18	1	13.18	76.23	<0.0001	***
C^2^	13.79	1	13.79	79.75	<0.0001	***
D^2^	6.16	1	6.16	35.61	<0.0001	***
Residual	2.42	14	0.1729			
Lack of Fit	2.05	10	0.2049	2.2	0.2324	Not significant
Pure Error	0.3723	4	0.0931			
Cor Total	41.35	28				
R^2^	0.9414		R^2^_Adj_	0.8829		
C.V.%	3.3353		Pred R-Squared	0.7005	A_deq_ Precision	12.8479

*** *p* < 0.001.

**Table 3:** Chemical characteristics of polysaccharides extracted with different methods from *D. huoshanense*.

	DHP-E	DHP-UM	DHP-H
Yield (%)	14.30 ± 0.25	12.61 ± 0.06	16.16 ± 0.31
Carbohydrate (%)	68.91 ± 0.32	71.19 ± 0.13	75.32 ± 0.02
Uronic acid (%)	4.18 ± 0.23	3.52 ± 0.11	4.80 ± 0.26
Protein (%)	13.08 ± 0.18	8.28 ± 0.21	7.17 ± 0.23
Total phenolics (mg GAE/g)	4.20 ± 0.23	1.56 ± 0.41	2.54 ± 0.31

**Table 4:** Weight-average molecular weight (Mw) and molar ratios of constituent monosaccharides of DHP.

	DHP-E	DHP-UM	DHP-H
Mw (Da)			
Fraction 1	6.92 × 10^5^	6.89 × 10^5^	5.64 × 10^5^
Fraction 2	9.63 × 10^4^	7.60 × 10^5^	4.82 × 10^5^
Fraction 3	2.12 × 10^4^	-	-
Fraction 4	2.63 × 10^4^	7.20 × 10^4^	4.98 × 10^4^
Fraction 5	5.23 × 10^4^	-	-
Monosaccharide composition (molar ratio, %)			
Man	68.49 ± 1.13	86.03 ± 0.81	85.22 ± 0.45
Glc	31.51 ± 1.13	13.97 ± 0.81	14.78 ± 0.45

**Table 5:** Methylation analysis of DHP.

Peak	RT	PMAA	Type of Linkage	Fragments (*m*/*z*)	DHP-E	DHP-UM	DHP-H
1	8.493	1,5-Di-O-acetyl-1-deuterio-2,3,4,6-tetra-O-methyl-D- glucitol	Glc*p*-(1→	43, 71, 87, 101, 117, 129, 145, 161	1.00	1.00	1.00
2	9.759	1,4,5-Tri-O-acetyl-1-deuterio-2,3,6-tri-O-methyl-D-mannitol	→4)-Man*p*-(1→	59, 71, 87, 102, 118, 129, 143, 162, 173, 189, 203	4.63	16.19	36.03
3	9.842	1,4,5-Tri-O-acetyl-1-deuterio-2,3,6-tri-O-methyl-D-glucitol	→4)-Glc*p*-(1→	59, 71, 87, 99, 118, 129, 142, 159, 173, 187, 203	2.51	5.37	12.57
4	11.211	1,4,5,6-Tetra-O-acetyl-1-deuterio-2,3-di-O-methyl-D-mannitol	→4,6)-Man*p*-(1→	59, 74, 85, 102, 118, 127, 142, 162, 171, 187, 201	0.14	0.31	0.18

## Data Availability

Data are contained within the article and Supplementary Materials.

## Supplementary Materials

The following supporting information can be downloaded at https://www.mdpi.com/article/10.3390/molecules28248019/s1, Figure S1. Effect of different (A) liquid–solid ratio, (B) extraction time, (C) extraction temperature, (D) microwave power, and (E) microwave minute on the yield of polysaccharides with UMAE. Response surfaces plots (F,H,J,L,N,P,R,T,V, and X) and contour plots (G,I,K,M,O,Q,S,U,W, and Y) showing the effects of liquid–solid ratio, extraction time, extraction temperature, microwave power, and microwave minute on the yield of polysaccharides with UMAE; Figure S2. Effect of different (A) extraction temperature, (B) extraction time, and (C) liquid–solid ratio on the yield of polysaccharides with HWE. Response surfaces plots (D,F, and I) and contour plots (E,G, and I) showing the effects of extraction temperature, extraction time, and liquid–solid ratio on the yield of polysaccharides with HWE; Table S1. Experimental design for independent variables and the response of the extraction yield of UMAE in Box–Behnken design; Table S2. ANOVA for response surface quadratic model for the yield of UMAE; Table S3. Experimental design for independent variables and the response of the extraction yield of HWE in Box–Behnken design; Table S4. ANOVA for response surface quadratic model for the yield of HWE.
