# Supplementary material for: Optimization of Three Extraction Methods and Their Effect on the Structure and Antioxidant Activity of Polysaccharides in Dendrobium huoshanense
Source: Molecules. 2023 Dec 8;28(24):8019. doi: 10.3390/molecules28248019 (PMC10745764; doi:10.3390/molecules28248019)
Supplement: Supplementary file 1 [file molecules-28-08019-s001.zip › molecules-2728362-supplementary.pdf]

molecules

MDPI

Article

Optimization of Three Extraction Methods and Their Effect on the Structure and Antioxidant Activity of Polysaccharides in *Dendrobium huoshanense*

Hua Zhu [1], Xin Yi [1], Si-Si Jia [1], Chun-Yao Liu [1], Zi-Wei Han [1], Bang-Xing Han [2], Gong-Cheng Jiang [3], Zheng-Feng Ding [3], Ren-Lei Wang [3,*] and Guang-Ping Lv [1,*]

[1] School of Food and Pharmaceutical Engineering, Nanjing Normal University, Nanjing 210046, China; wfzh8233@163.com (H.Z.); 212702035@njnu.edu.cn (X.Y.); 18835540923@163.com (S.-S.J.); wissingni@163.com (C.-Y.L.); hinziwei@163.com (Z.-W.H.)

[2] College of Biological and Pharmaceutical Engineering, West Anhui University, Lu'an 237012, China; hanbx1978@wxc.edu.cn

[3] Key Laboratory of Biological Functional Molecules of Jiangsu Province, College of Life Science and Chemistry, Jiangsu Second Normal University, Nanjing 211200, China; ji680103@163.com (G.-C.J.); ding@jssnu.edu.cn (Z.-F.D.)

* Correspondence: wrl@jssnu.edu.cn (R.-L.W.); guangpinglyu@njnu.edu.cn (G.-P.L.)

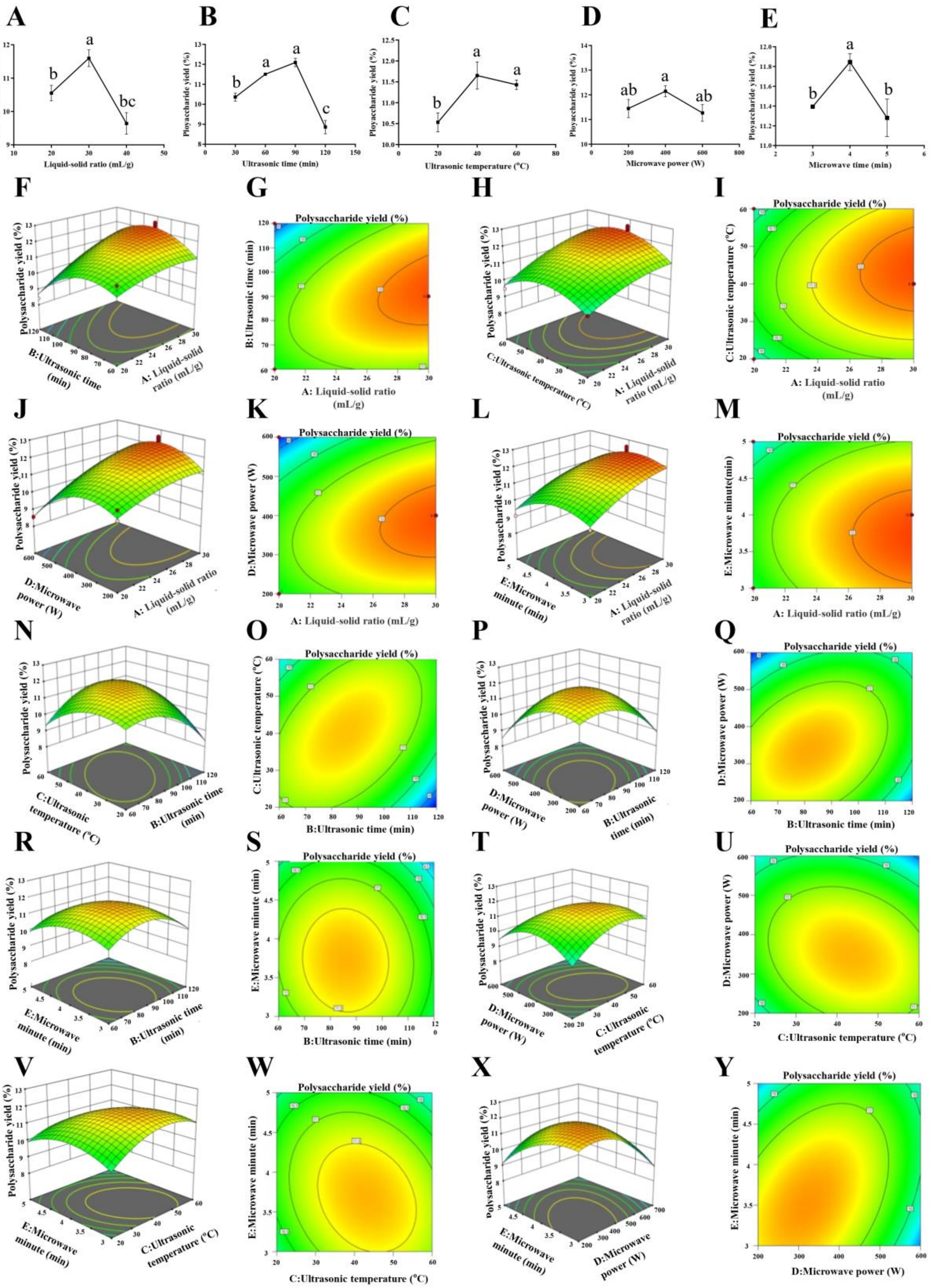

Figure S1. Effect of different (**A**) liquid-solid ration, (**B**) extraction time, (**C**) extraction temperature, (**D**) microwave power and (**E**) microwave minute on the yield of polysaccharides by UMAE. Response surfaces plots (**F**, **H**, **J**, **L**, **N**, **P**, **R**, **T**, **V** and **X**) and contour plots (**G**, **I**, **K**, **M**, **O**, **Q**, **S**, **U**, **W** and **Y**) showing the effects of liquid-solid ration, extraction time, extraction temperature, microwave power and microwave minute on the yield of polysaccharides by UMAE. Significant ($p < 0.05$) differences are shown by data bearing different letters (a–c).

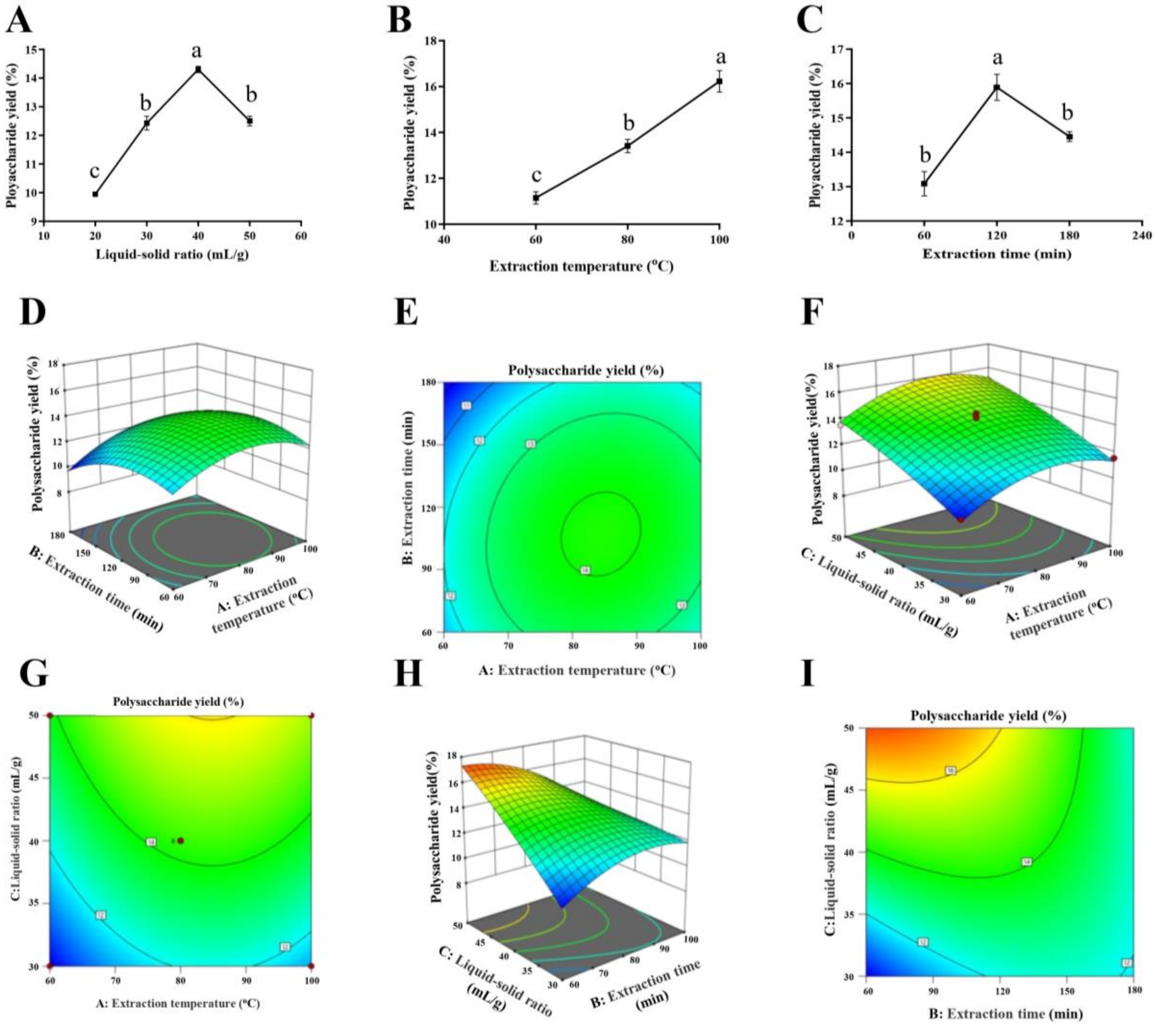

Figure S2. Effect of different (**A**) extraction temperature, (**B**) extraction time and (**C**) liquid-solid ration on the yield of polysaccharides by HAE. Response surfaces plots (**D**, **F** and **H**) and contour plots (**E**, **G** and **I**) showing the effects of extraction temperature, extraction time and liquid-solid ratio on the yield of polysaccharides by HAE. Significant ($p < 0.05$) differences are shown by data bearing different letters (a–c).

Table S1 Experimental design for independent variables and the response of the extraction yield of UMAE in Box-Behnken design.

Run	Coded variables					Uncoded variable					Response
	A	B	C	D	E	A (Liquid–solid ratio, (mL/g))	B (Ultrasonic time, min)	C (Ultrasonic temperature, °C)	D (Microwave power, W)	E (Microwave minute, min)	Y (the extraction yield of DHP %)
1	-1	1	0	0	0	20:1	120	40	400	4	8.42
2	0	1	-1	0	0	30:1	120	20	400	4	9.24
3	0	0	0	1	1	30:1	90	40	600	5	10.16
4	-1	-1	0	0	0	20:1	60	40	400	4	11
5	0	0	-1	0	1	30:1	90	20	400	5	10.29
6	0	1	0	-1	0	30:1	120	40	200	4	9.66
7	0	-1	1	0	0	30:1	60	60	400	4	8.92
8	0	-1	0	0	1	30:1	60	40	400	5	9.74
9	0	0	0	0	0	30:1	90	40	400	4	11.79
10	0	0	0	1	-1	30:1	90	40	600	3	9.66
11	0	0	1	0	0	40:1	90	60	400	4	10.86
12	0	-1	0	0	-1	30:1	60	40	400	3	10.03
13	0	1	0	0	-1	30:1	120	40	400	3	11.42
14	0	0	0	0	0	30:1	90	40	400	4	12.32
15	0	1	0	1	0	30:1	120	40	600	4	10.77
16	0	0	0	0	-1	40:1	90	40	400	3	11.4
17	0	0	0	-1	1	30:1	90	40	200	5	8.64
18	0	0	1	-1	0	30:1	90	60	200	4	11.62
19	0	-1	0	-1	0	30:1	60	40	200	4	10.81
20	0	-1	0	0	0	40:1	60	40	400	4	9.18
21	0	0	-1	0	-1	30:1	90	20	400	3	10.79
22	0	0	0	-1	-1	30:1	90	40	200	3	11.32
23	0	-1	-1	0	0	30:1	60	20	400	4	10.66
24	0	0	-1	-1	0	30:1	90	20	200	4	9.43
25	0	1	0	0	1	30:1	120	40	400	5	10.75

26	0	0	1	0	1	30:1	90	60	400	5	10.51
27	-1	0	-1	0	0	20:1	90	20	400	4	9.79
28	-1	0	0	0	1	20:1	90	40	400	5	9.07
29	0	0	0	0	0	30:1	90	40	400	4	12.5
30	0	1	0	0	0	40:1	120	40	400	4	10.74
31	0	0	1	1	0	30:1	90	60	600	4	10.22
32	0	0	0	0	1	40:1	90	40	400	5	10.25
33	0	0	-1	1	0	30:1	90	20	600	4	9.93
34	-1	0	0	0	-1	20:1	90	40	400	3	10.09
35	0	0	-1	0	0	40:1	90	20	400	4	9.66
36	-1	0	1	0	0	20:1	90	60	400	4	9.49
37	-1	0	0	-1	0	20:1	90	40	200	4	10.22
38	0	-1	0	1	0	30:1	60	40	600	4	9.09
39	0	0	0	-1	0	40:1	90	40	200	4	9.7
40	-1	0	0	1	0	20:1	90	40	600	4	8.56
41	0	0	0	0	0	30:1	90	40	400	4	12.39
42	0	1	1	0	0	30:1	120	60	400	4	11.52
43	0	0	0	0	0	30:1	90	40	400	4	11.88
44	0	0	0	1	0	40:1	90	40	600	4	10.48
45	0	0	0	0	0	30:1	90	40	400	4	12.57
46	0	0	1	0	-1	30:1	90	60	400	3	12.19
47	-1	0	0	-1	0	20:1	90	40	200	4	10.78
48	0	1	0	1	0	30:1	120	40	600	4	11.08
49	0	1	1	0	0	30:1	120	60	400	4	11.42
50	0	0	-1	0	-1	30:1	90	20	400	3	9.97

Table S2 ANOVA for response surface quadratic model for the yield of UMAE.

Source	Sum of squares	Df	Mean square	F-value	p-Value	
Model	51.49576	20	2.574788	13.65901	7.76E-10	significant
A-Liquid-solid ratio	16.0156	1	16.0156	84.96129	4.05E-10	***
B-Ultrasonic time	0.882579	1	0.882579	4.681999	0.038857	*
C-Ultrasonic temperature	0.216094	1	0.216094	1.146356	0.293144	
D-Microwave power	2.821115	1	2.821115	14.96575	0.000571	***
E-Microwave minute	1.511223	1	1.511223	8.016898	0.008336	**
AB	4.2849	1	4.2849	22.731	4.84E-05	***
AC	0.5625	1	0.5625	2.98401	0.094728	
AD	2.503331	1	2.503331	13.27994	0.001041	**
AE	0.004225	1	0.004225	0.022413	0.882029	
BC	4.124853	1	4.124853	21.88196	6.21E-05	***
BD	2.932033	1	2.932033	15.55416	0.000466	***
BE	0.0361	1	0.0361	0.191507	0.664907	
CD	0.9025	1	0.9025	4.787679	0.036869	*
CE	0.701478	1	0.701478	3.721277	0.063561	
DE	2.5281	1	2.5281	13.41134	0.000993	***
A^2	15.35217	1	15.35217	81.44183	6.42E-10	***
B^2	10.92444	1	10.92444	57.95311	2.16E-08	***
C^2	7.153454	1	7.153454	37.94841	1.03E-06	***
D^2	12.56768	1	12.56768	66.67036	5.28E-09	***
E^2	5.145611	1	5.145611	27.29699	1.36E-05	***
Residual	5.466637	29	0.188505			
Lack of Fit	4.383104	20	0.219155	1.820338	0.178664	not significant
Pure Error	1.083533	9	0.120393			
Cor Total	56.96239	49				
R^2	0.9040		R^2_{Adj}	0.8378		
C.V.%	4.1509		Pred R-Squared	0.6803	A_{deq} Precision	13.1120

* $p < 0.05$; ** $p < 0.01$; *** $p < 0.001$.

Table S3 Experimental design for independent variables and the response of the extraction yield of HWE in Box-Behnken design.

Run	Coded variables			Uncoded variable			Response
	A	B	C	A (Extraction temperature, °C)	B (Extraction time, min)	C (Liquid-solid ratio, (mL/g))	Y (the extraction yield of DHP %)
1	-1	0	1	60	120	50:1	13.54
2	0	1	-1	80	180	30:1	11.17
3	-1	0	-1	60	120	30:1	10.08
4	1	0	-1	100	120	30:1	11.57
5	0	0	0	80	120	40:1	14.71
6	0	0	0	80	120	40:1	14.32
7	1	0	1	100	120	50:1	15.03
8	0	-1	1	80	60	50:1	17.86
9	0	1	1	80	180	50:1	12.05
10	0	-1	-1	80	60	30:1	10.01
11	1	1	0	100	180	40:1	12.03
12	-1	-1	0	60	60	40:1	11.87
13	1	-1	0	100	60	40:1	12.59
14	-1	1	0	60	180	40:1	10.05
15	0	0	0	80	120	40:1	14.52
16	0	0	0	80	120	40:1	14.11
17	0	0	0	80	120	40:1	14.6
18	0	0	0	80	120	40:1	13.98
19	0	0	0	80	120	40:1	14.34
20	0	0	0	80	120	40:1	13.94

Table S4 ANOVA for response surface quadratic model for the yield of HWE.

Source	Sum of squares	Df	Mean square	F-value	p-Value	
Model	75.37	9	8.37	51.12	< 0.0001	significant
A	4.03	1	4.03	24.62	0.0006	***
B	6.18	1	6.18	37.71	0.0001	***
C	30.62	1	30.62	186.88	< 0.0001	***
AB	0.3969	1	0.3969	2.42	0.1506	
AC	0.0000	1	0	0	1	
BC	12.15	1	12.15	74.14	< 0.0001	***
A^2	9.59	1	9.59	58.57	< 0.0001	***
B^2	6.93	1	6.93	42.3	< 0.0001	***
C^2	0.4429	1	0.4429	2.7	0.1312	
Residual	1.64	10	0.1638			
Lack of fit	1.06	3	0.3545	4.32	0.0508	not significant
Pure error	0.5748	7	0.0821			
Correlation total	77.01	19				
R^2	0.9787		R^2_{Adj}	0.9599		
C.V.%	3.0853		Pred R-Squared	0.95958206909385	A_{deq} Precision	26.6202

***$p < 0.001$.
